# Barriers to and enablers of availability and integration of palliative care into routine services at Charlotte Maxeke Johannesburg Academic Hospital, South Africa

**DOI:** 10.1186/s12904-025-01778-3

**Published:** 2025-06-02

**Authors:** Sukoluhle Pilime, Mpho Ratshikana, Oludoyinmola Ojifinni, Latifat Ibisomi

**Affiliations:** 1 School of Public Health Sciences, Faculty of Health, University of Waterloo, Waterloo, Canada; 2 Wits Centre for Palliative Care, Faculty of Health Sciences, University of the Witwatersrand, Johannesburg, South Africa; 3 School of Clinical Medicine, Faculty of Health Sciences, University of the Witwatersrand, Nigerian Institute of Medical Research, Lagos, Nigeria; 4 Division of Epidemiology & Biostatistics, School of Public Health, University of the Witwatersrand, Nigerian Institute of Medical Research, Lagos, Nigeria

**Keywords:** Palliative care, CFIR, Barriers, Enablers, Integration

## Abstract

**Background:**

The prevalence of chronic diseases among the South African population is increasing. The incidence of cancers is high and expected to double by 2030 while the HIV burden is disproportionately high. These are indicators of the need for palliative care services in the South African health system. While some palliative care service exists, there are challenges with availability and integration into routine services nationally. In Gauteng Province, CMJAH is one of two quaternary hospitals that provide palliative care services. We sought to find the barriers and enablers for the availability and integration of palliative care into routine services at CMJAH.

**Methods:**

We used an exploratory qualitative research approach. Study sites were the internal medicine, surgical, and medical oncology departments, the palliative care unit, and the administration block at CMJAH. We interviewed four groups of personnel: the management of the four departments, previous and current staff in the palliative care unit, and hospital management staff. A hybrid of inductive and deductive coding was conducted, and the final codebook was developed using all the domains and selected constructs of the Consolidated Framework for Implementation Research (CFIR).

**Results:**

Barriers and enablers were categorised according to the domains of the CFIR. We found that individual characteristics such as knowledge and awareness of palliative care, adequate training, and positive attitude towards palliative care encourage the availability of services at a hospital. The availability of a budget for palliative care, a conducive environment for service provision, and management buy-in are also key determinants for palliative care. Poor planning, lack of implementation of the national policy framework and strategy for palliative care, and over-reliance on external donor funds are some of the hinderances for availability and integration of palliative care at a public hospital.

**Conclusion:**

Despite the importance of palliative care, implementation is a challenge at CMJAH. The availability of a palliative care budget from relevant Government departments, and inclusion of some roles (navigators, spiritual chaplains) in the personnel structure for the Health Department are some of the ways through which availability and integration of the service can be improved across public hospitals in South Africa.

## Background

By the year 2030, Non-Communicable Diseases (NCDs) will account for the majority of deaths in developing countries, and cancer-related illnesses will reach approximately 1.28 million new cases [1]. These projections are attributable to the adoption of risk prone lifestyles, increasing aging populations, deficient diagnostics, and sub-standard preventive and curative treatment services [1]. The WHO Africa region falls behind in human development and health services compared to other regions [2], and HIV/AIDS continues to pose the greatest burden of disease [3]. Moreover, there has been a steep increase in chronic and communicable diseases in the African region, creating challenges in healthcare service delivery, especially among deeply impoverished populations [2]. Based on this, there is a critical need for palliative care service provision in developing countries, particularly in Sub-Saharan Africa [3]. The WHO estimates that approximately ten million people in Africa require palliative care services annually [1], yet in most African countries, there are less than two palliative care services available per one million people [4].

Palliative care is defined as the management of the physical, psychosocial, and spiritual suffering of patients who have been diagnosed with life-limiting or life-threatening illnesses [5]. The provision of palliative care is vital in ensuring pain and symptom relief to patients who suffer from diseases such as AIDS, cancer, diabetes, and chronic respiratory and cardiovascular diseases [5], which are prevalent in Africa. In South Africa, this specialised care is particularly crucial due to the country’s disproportionately high HIV/AIDS burden, estimated to be 9% of the total world population [6]. The incidence of breast, cervical, prostate, and paediatric cancers is reported to be high in the country and is expected to have doubled by the year 2030 [7]. A study conducted in 2011 found that at the time South Africa had 160 palliative care services, which supported only 40,000 people [6], against an estimated need of about 310,800 people, based only on mortality data [8].

South Africa is a signatory of the World Health Assembly Resolution 67.19 on “Strengthening of palliative care as a component of comprehensive care throughout the life course” [9]. The country has also developed a National Policy Framework and Strategy on Palliative Care (NPFSPC), yet there are still challenges in policy implementation at a national level [10]. Hospital-based palliative care services in the country are rare and most services are provided at the community level [11].

In Gauteng, South Africa’s largest province, public hospital-based palliative care services are offered at Chris Hani Baragwanath Academic Hospital (CHBAH) and Charlotte Maxeke Johannesburg Academic Hospital (CMJAH) [12]. Hospital-based day-to-day consult services are provided by a Multidisciplinary Team (MDT) consisting of clinicians (doctors and professional nurses), social workers, chaplains, and patient navigators who are housed in the palliative care unit (PCU) within the hospitals. Support staff including a Project Director, Project Manager, Monitoring and Evaluation Manager, Project Administrator, and IT staff are available, and some of these positions are funded through external sources. While palliative care is established and is being provided at CHBAH [13], services are still heavily dependent on donor funding and have not been fully integrated at CMJAH [14].

According to guidance from the NPFSPC, fully integrated palliative care in a tertiary hospital includes care provided from diagnosis to death, seamlessly embedded across hospital services through an MDT approach [10]. The policy shifts from the “old concept” (end of life only) to the “new concept” (from diagnosis), ensuring early access to palliative care [10]. Literature has also shown a fully integrated palliative care approach to include dedicated palliative care units or beds for complex cases, symptom management and family support, structured referral pathways to palliative care within the hospital, and linkages to community services on discharge [15]. Dedicated funding from the Department of Health, which ensures human resources and infrastructure availability, and a robust monitoring and evaluation system enables the integration of palliative care into routine services [10].

CMJAH is classified as a central hospital, providing “highly specialised health care services and serving as a specialist referral centre for tertiary hospitals in Gauteng and other provinces in South Africa” [16]. The hospital is one of ten central hospitals in the country and has a bed capacity of 1,088 beds; over 4,000 professional and support staff and is Gauteng’s main oncology hospital [13]. Despite the inordinate need, there are still challenges with availability, integration, and routinisation (adoption of palliative care services into daily practice) of palliative care [14]. The objectives of this study were to describe the barriers to and enablers for the availability and integration of palliative care services into routine services at CMJAH.

## Methods

### Design

We used an exploratory qualitative approach, guided by the Consolidated Framework for Implementation Research framework. The CFIR framework provides a guide to systematically assessing implementation contexts at different levels, and identifying perceived factors that might affect the implementation and effectiveness of interventions [17]. This approach was considered the most appropriate to describe the barriers to and enablers for palliative care availability and integration from the perception of the personnel at the hospital and developing recommendations for implementation.

Conducting this study, we recognised the importance of our collective positionality and reflexivity to ensure the trustworthiness of our findings. The team of researchers consisted of palliative care practitioners and implementation scientists: MR and OO are medical doctors, and MR is a palliative care specialist. SP is an epidemiologist and has received training in palliative care programming. LI is a professor and specialist in implementation science. This research team views palliative care availability and integration at CMJAH as a multifaceted reality, shaped by institutional administrative, clinical, and socio-cultural dynamics. Epistemologically, we acknowledged the situated knowledge of the CMJAH staff, and we prioritised contextual knowledge co-constructed through interviews with the multi-disciplinary staff involved in patient care at various levels at the hospital. With the combined experience, knowledge, and training in palliative care of the research team, we identified data segments crucial for the study and gave meaning to the collected data. By critically reflecting on our values, realities, and knowledge-building, we ensured findings authentically reflected CMJAH’s palliative care services landscape, balancing advocacy with neutrality for trustworthy results.

### Study site

This study was conducted at the Charlotte Maxeke Johannesburg Academic Hospital (CMJAH) in South Africa. CMJAH is a public central hospital, mandated to provide a full range of services, including quaternary, tertiary, and secondary services [18]. The hospital also serves as a referral hospital for other lower-level hospitals within and around Gauteng province [18]. Within CMJAH, the selected study sites for this research were the Palliative Care Unit, the Internal Medicine, Radiation Oncology, Medical Oncology, and Surgical Departments. These departments manage patients who suffer from chronic, life-threatening illnesses, and require palliative care services. The hospital management is housed within the administration block, and it was also included as a study site.

### Study population

The study population included personnel from the following departments: Internal Medicine, Radiation Oncology, Medical Oncology, Surgery, Palliative Care, and hospital administration. Personnel from the palliative care team consisted of individuals previously involved in providing palliative care services at the hospital and personnel currently providing services.

### Sampling criteria

We predetermined the departments from which we would sample participants and then purposively selected study participants from these departments. We projected that data saturation would be achieved with a sample of 10–15 participants.

Members of management from selected departments were chosen as they were best suited to provide a management perspective of the routine operations in the selected departments. Participants were selected from the palliative care unit to give accounts of their experiences with palliative care service provision in the hospital. The central hospital management, responsible for overall hospital governance and collectively overseeing the hospital’s strategic and operational functions, including issues related to the availability and integration of palliative care, was also included in the study.

Participant selection using this criterion was done to acquire data from all relevant entities within the hospital. Though the participants were purposively sampled, the total number of participants in each department was determined upon reaching data saturation. We interviewed 12 participants from the four subcategories. This included four members of management from the selected departments, six personnel from the palliative care unit (three previous and three current), and two personnel from hospital management. Table 1 shows the distribution of the study population and the themes that were explored with each group of participants.

**Table 1:** Categorisation of participants and themes explored

Study population categories	Number of participants interviewed per category	Themes that were explored
1. Members of management (Medical Oncology, Radiation oncology, Surgery, Internal Medicine)	4	Knowledge about the palliative care package, perception about integrating palliative care into routine services, prioritization of palliative care services, buy-in towards provision of palliative care in departments
2. Hospital management	2	Perception about availability of funds for implementation, knowledge about policy and guidelines, prioritization of palliative care services, awareness of palliative care programs at other hospitals
3. Previous palliative care programme staff	3	Perception of barriers, perception of enablers, insights into previous failures and successes
4. Current palliative care programme staff	3	Awareness of previous failures, awareness of previous successes, perceptions towards the uptake of the program

### Data collection

In-depth interviews were conducted through the administration of open-ended, semi-structured interview guides. The interview guides were patterned around the Consolidated Framework for Implementation Research (CFIR) domains [17]. To test the tool and adjust it for greater understanding, the data collection tools were pretested at Helen Joseph Hospital with two people of similar relevance as the intended study population.

Interviews lasted between 30 and 45 min and were recorded using a portable device with a cell phone as backup. The data were collected between the period of March 2023 and August 2023. We reached data saturation after having interviewed 12 participants from the selected departments. We concluded that we had reached saturation after observing redundancy in the collected data with no new codes emerging from the transcripts. The investigators chose the palliative care unit as the first department from which participants would be interviewed. Initially, two participants were selected from each subgroup (previous and current staff), but after analysing the data, the researchers observed the need to increase the sample size by an additional participant for both groups, after which point saturation was achieved. From the initial interviews with this group, themes related to the referral process from the previous and current palliative care teams were not fully described hence the decision to seek further insights by an additional participant in each group. The same process for sampling and data saturation was repeated for the remainder of the categories.

### Data management and analysis

Audio recordings were transcribed verbatim using Otter software, and the NVivo 14 software was used to manage and analyse data.

For this research, we used a hybrid of inductive and deductive coding [19] to develop codes from the collected data. We used inductive coding to develop open codes from the interview transcripts and then followed up by comparing the codes for similarities and differences, and labelling similarities in the data with the same code. Inter-coder reliability (measurement of agreement for code development between two different coders) [20] was achieved by having an independent researcher who was not a member of the research team code three of the twelve transcripts, assessing for similarities and differences between the codes, and then reaching a consensus on the final codebook for the inductive coding process.

**Fig. 1:**
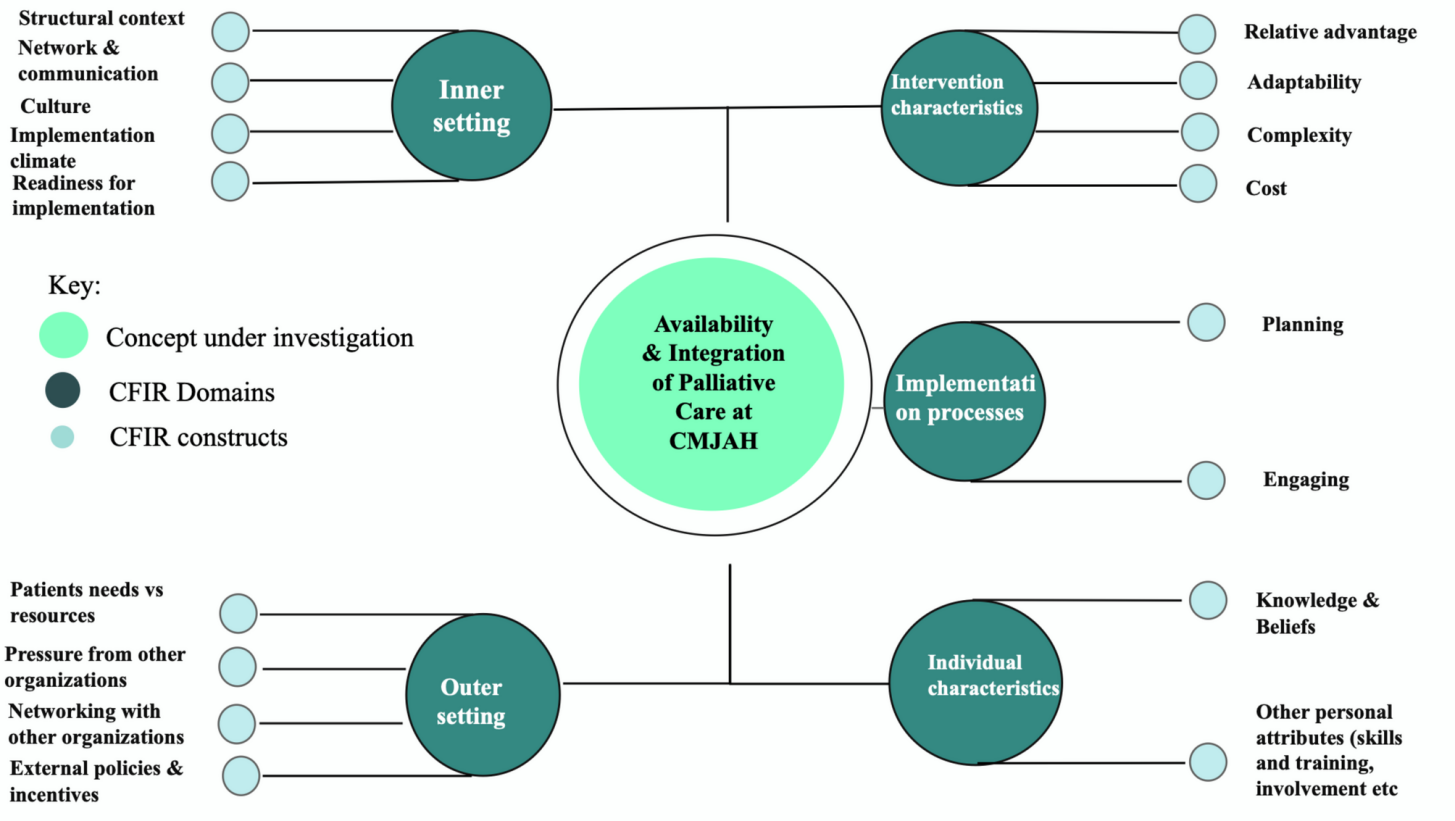
Conceptual map of the categories and codes developed according to the CFIR Framework

For the deductive coding process, we grouped the codes identified during the inductive coding process under domains and constructs of the CFIR framework. Data were collected and findings were assigned to the five domains of the framework, which are: Individual characteristics (e.g., knowledge and beliefs, other personal attributes, i.e., skills and qualifications); Intervention characteristics (e.g., adaptability, complexity, cost, relative advantage); Implementation process (e.g., planning, engaging); Inner setting (e.g., structural context, network and communication, culture, implementation climate, readiness for implementation); Outer setting (e.g., a patient needs vs. resources, networking, external policies, and incentives, pressure from other organisations). We then identified codes not captured by the framework but had been identified during the inductive coding process. A conceptual map of the domains and selected constructs is shown in Fig. 1.

We investigated the relationships between the concept of interest (availability and integration of palliative care at CMJAH), and the developed codes, guided by the domains and constructs of the CFIR. The codes were then separated into either barriers or enablers. Throughout the process, memos were written to keep track of the analytical process.

### Ethical considerations

Eligible staff were presented with information sheets summarising the study and were requested to sign a written informed consent form for participating in the study and for audio recordings of their interviews. Permission to conduct the study from the hospital management was obtained after submitting approval letters from the Heads of each department where the study was going to be conducted. Ethical clearance to conduct the study was received from the Wits Human Research Ethics Committee (HREC) [ethics number M220147].

## Results

The characteristics of the healthcare professionals interviewed for this study are shown in Table 2. There is some overlap in categorisation as some of the medical doctors also doubled as members of management.

**Table 2:** Characteristics of research participants

Category	Number of participants (*n*)
**Sex**	
Male	3
Female	9
**Departments**	
Internal Medicine	1
Medical oncology	1
Radiation oncology	1
Surgery	1
Palliative Care Unit: *Current staff* *Previous staff*	3 3
Management	2
**Type of healthcare professional**	
Clinical Nurses	3
Medical Doctors	6
Members of management from selected departments	3
Nursing Managers	3

The identified barriers and enablers were categorised according to the domains of the CFIR framework. For this study, we utilised all the domains of the CFIR framework, however, we selected constructs that were relevant to answer our research question. We found that sustainability was an emerging issue from the transcripts but is not covered as an independent construct on the CFIR. From the literature search, we established that sustainability is measured under the inner and outer setting domain on the CFIR [21], and we coded issues arising under sustainability as such. Figure 1 illustrates the conceptual map of the domains and identified constructs that were utilised for this study.

Table 3 is a presentation of the identified barriers and enablers for availability and integration of palliative care at CMJAH presented according to the five domains of the CIFR framework and related sub-constructs.

**Table 3:** Barriers and enablers for availability and integration of palliative care at CMJAH

CFIR Framework constructs and sub-constructs	Enablers	Barriers
**Individual characteristics**		
*Knowledge & Beliefs*	• Adequate knowledge of the components of palliative care among all interviewed personnel • Knowledge of previous palliative care project run at the hospital • Management awareness of challenges at the palliative care unit	• Insufficient knowledge by some health care providers leading to late referrals • Insufficient knowledge of the palliative care screening tool leading to non-use
*Other personal attributes (Skills and Training)*	• Palliative care training received by all staff members from the palliative care unit • Over 1 year of experience providing palliative care services among health care professionals at the palliative care unit • Routine education sessions conducted in the wards by palliative care staff • Adequate skills to provide palliative care among staff	• Some staff members from the different departments within the hospital have not received palliative care training • Palliative care not embedded as part of training for medical oncologists
***Intervention characteristics***		
*Relative advantage*	• Reduced hospitalisation among patients receiving palliative care services • Improved patient outcomes among patients receiving palliative care services	• Increased staff workload for palliative care staff • Additional tasks of screening patients for eligibility for palliative care
*Adaptability*	• With adequate training, health care providers from the different wards can incorporate palliative care into their routine work schedules. • The hospital academic meeting can be adapted to include presentations from palliative care	• Poor administration support from management for implementation • Previous palliative care project not part of the oncology clinic
*Complexity*	• Perception by management that palliative care can be implemented • Perception by health care providers that it is not difficult to incorporate palliative care clinical care into general practice • Ease of inclusion of palliative care into standard care	• Palliative care unit far from the oncology clinic (no way of tracking if referred patients make their way to the palliative care unit) • Some staff in the palliative care unit responsible for other tasks within the hospital
*Cost*	• Funding from Bristol Myers Squibb Foundation (BMSF) • Hospital provision of clinicians based at the palliative care unit (3 nurses & 1 doctor)	• No allocated budget for Human Resources, IT support, financial, and allied support • No solid budget for palliative care at the hospital
***Implementation process***		
*Planning (steps for effective implementation)*	• Possible for palliative care to be included in the ward rounds • Education sessions conducted among health care providers in wards to improve referral and identification of palliative care patients • Planning to conduct awareness sessions throughout the departments in the hospital	• No set procedures and Standard Operating Procedures for palliative care implementation • Need for improvement of the referral system
*Engaging*	• Palliative care has been accepted in some departments • CMJAH clinical director is actively involved in palliative care • Launch of palliative care services conducted, which was inclusive of other hospitals and had representation from all departments	• Inadequate engagement with other disciplines to discuss patient cases • Palliative care is not involved during ward rounds as with other specialties such as haematology, microbiology, etc.
***Inner setting***		
*Structural context*	• Available capacity for palliative care services due to the nature of health care facility • Support from management for the currently externally funded project to be run in the hospital	• Central hospital with high patient volumes which would require more human resources to provide palliative care • Poor visibility of palliative care in the hospital wards, especially in oncology departments to enable referrals and patient uptake.
*Network and communication*	• Some level of communication between the palliative care department and the other hospital wards, though this can be improved	• Incapacity of palliative care personnel to be active in all the wards daily • Poor communication between departments
*Implementation climate*	• Enthusiasm for personnel who have bought into palliative care • Management acceptance of the project creates motivation for staff to implement	• Palliative care not well supported and not well encouraged– very individual-driven • High turnover of doctors, resulting in discontinuation of services when trained doctors leave • Resistance to prescribe opioids for pain by some doctors
*Culture*	• Value placed on palliative care among other disciplines and departments	• Inadequate attention given for non-clinical allied services e.g. spiritual services and patient navigation
*Readiness for implementation*	• Already existing section allocated for implementation of services	• No set standard operating procedures for implementation • Inadequate staff for providing allied services • Non-recognition of palliative care as a sub-department resulting in challenges with patient registration and ultimately provision of services
***Outer setting***		
*Patient needs vs. resources*	• Allocation of additional nurses for palliative care	• Overreliance on donor funds • No budget for palliative care from Gauteng Department of Health (GDoH) • No budget for palliative care from the hospital
*Networking with other organisations*	• Some engagements have been made with the National Department of Health (NDoH) and hospices within the province • Community-level training for palliative care conducted	• Poor referrals from other district hospitals
*External policies and incentives*	• Availability of the National Policy and Framework for palliative care • Awareness of the policy among management	• Palliative care roles such as spiritual counsellors and patient navigators are not part of the Department of Health (DoH) structure
*Pressure from other organisations*	• Established services, with a published model of care, provided at another tertiary institution in the province (Chris Hani Baragwanath Academic Hospital)	• Inadequate coordination among the relevant stakeholders for palliative care between the two tertiary hospitals in the province

The data were disaggregated into two categories: either barriers or enablers and the results are presented according to this disaggregation, starting with the enablers.

## Enablers

From the interviews conducted, the researchers identified the following enablers for the availability and integration of palliative care services at CMJAH, categorised according to the domains and selected constructs of the CFIR framework.

### Domain 1: individual characteristics

#### Knowledge

All participants interviewed for this study were aware of the palliative care services provided at CMJAH. Moreover, they were only aware of the components of palliative care that involve providing holistic support and addressing total pain for terminally ill patients. This was observed from all the individuals who were interviewed for this research.

*“I understand that it’s the care for patients who are terminally ill. It’s for patients who have been diagnosed with a disease that is uncurable. And so in palliation*,* we provide patients care in terms of their spiritual care*,* physical*,* emotional*,* psychological*,* and social care. That’s what the holistic approach is what palliative care is.”-***HD1**.

#### Skills and training

The palliative care team directly providing services at CMJAH consists of clinicians (1 Doctor and 3 professional nurses), a social worker and social auxiliary worker, two spiritual counsellors, and a patient navigator. We found that all members of the team received a specialised palliative care training during induction, in addition to their already existing qualifications. This training is facilitated through the Gauteng Centre of Excellence for Palliative Care and is conducted over five days, followed by practical training at a tertiary hospital. In addition to this training, at the time of the interviews, each member of the palliative care team had acquired over a year’s experience providing services. The following comments were made regarding the content of the training:

*“The training covered the definition of palliative care in the hospital*,* in the community*,* and the clinic. We were taught how to do the holistic approach to managing pain*,* meaning not just dealing with the physical pain*,* but assessing the patient holistically*,* that is physically*,* psychologically*,* spiritually*,* and socially.”*– **CN1**.

### Domain 2: intervention characteristics

#### Stakeholders’ perceptions about the relative advantage of implementing palliative care

The management and staff at CMJAH highlighted the advantages of palliative care services related to the early discharge of patients, resulting in the freeing of beds and reduction of costs associated with hospitalization.

*“By incorporating palliative care*,* we can discharge patients earlier*,* and so by not extending their hospital stay; we are actually helping the hospital financially*,* so I think it does more good than harm.”*– **MD1**.

Another participant also highlighted that with the availability of palliative care, fewer admissions are done in the hospital, resulting in more bed availability.

*“…. now they (patients) understand that not each and everything that they develop needs to bring them to the hospital*,* but a phone call to palliative care can really assist them. That will reduce the re-admission of some patients…”*– **CN2**.

*“You find that the patient doesn’t have physical problems that really warrant a hospital admission but because their emotional and social problems are not effectively addressed*,* they end up manifesting physical problems that lead to the patient being admitted. So*,* if palliative care services can be effectively integrated*,* I think we will have fewer admissions whereby we will be even saving a lot of money that we are spending in the hospital…”*- **NM1**.

#### Adaptability

The interviews revealed the potential for the selected departments to adapt their services towards integrating palliative care. Though having an in-house palliative care team within each department is not feasible, referrals can be made to the palliative care unit for the patients to receive relevant care.

*“… we screen patients ourselves in the wards and get referrals from various departments as well. The palliative team goes out to see those referrals and screening those patients almost on a daily basis*,* or if not at least three times a week*,* they go out into the wards and screen and counsel those patients. And then in terms of outpatient services*,* the teams go to the medical oncology clinics*,* as well as radiation oncology clinics to screen for patients there as well…”–***MD1**.

The same applies to training; although some clinicians within the department cannot be trained in palliative care, they can attend informal awareness creation sessions on how to identify patients who require it and provide relevant care.

*“…I think palliative care teams can give the initial education and training. But if you do it in a proper way*,* then you don’t need to call palliative care every single time. You can get them to teach you to advise*,* supervise*,* but it shouldn’t be about them doing all the work. It’s about teaching what they do. So that the next time you don’t need to call palliative care that becomes part of how you manage patients as well…”-***HD2**.

### Domain 3: implementation processes

#### Engaging

The clinical personnel from the departments selected for this study engage with the palliative care team by referring patients for palliative care.

*We have a palliative care team that sits in the clinic every day and facilitates us being able to refer patients to them. It also helps move the continuum of care. As we come sort of to the tail end of that active treatment*,* patients need more and more pain control more and more assistance on a supportive and holistic as well as a counselling point of view -***HD3**.

We found that the clinical director at the hospital is actively involved in activities at the palliative care unit. In addition, palliative care services have been officially launched at the hospital, and during this launch, there was representation of all the departments within the hospital and other hospitals within the province.

*“Our clinical director is quite involved with the palliative care project*,* which is funded through the BMS foundation. So from that point of view*,* there’s been a strong push from management to see this program succeed. So I think from their point of view*,* we do have management support.”–***HD2**.

### Domain 4: inner setting

#### Structural context

CMJAH is a central hospital, which provides highly specialised services for patients. This implies capacity for the provision of palliative care services at the hospital. There is a section in the hospital that has been allocated for palliative care services and active efforts are being made to ensure that palliative care is a sub-department or division in the hospital.

*“I’m actively working on it; actively working on getting palliative care as a sub-department division in this hospital*,*”–***HD2**.

#### Implementation climate

The hospital as an institution can provide palliative care services to patients through already existing systems that promote the availability of services. These include an allocated space for the palliative care department and buy-in from management. The interviewed palliative care staff reported that they have acquired visibility within the wards and created relationships with clinical personnel from the different departments, so they do not have to wait for referrals.

*“What exists now and makes the program to be successful*,* it’s that we don’t wait for the referrals. We don’t wait for the doctors to refer the patient to us*,* we go out there ourselves to screen the patients*,* especially in the medical oncology and radiation oncology wards…”*– **MD1**.

### Domain 5: outer setting

#### Networking with other organisations

Spiritual and patient navigation services are not part of the personnel structure that is currently in place in the Gauteng Department of Health (GDoH). Submissions have been made to the GDoH to include these roles in the hospital staff structure and are awaiting approval.

*“We need approval (from the Department of Health) of the personnel structure or the human resources for palliative care. We also need more nurses*,* spiritual counsellors*,* and social workers”*– **NM2**.

#### External policies and incentives

Personnel interviewed for this research were aware of the local policies that are in place for the implementation of palliative care in the hospital such as the National Policy Framework and Strategy for Palliative Care. Through the donor-funded palliative care centre, collaborations have been made to assist in the implementation of the policy in Gauteng province. A provincial team, comprising key personnel from the Gauteng Department of Health (GDoH) has been formed, and district task teams for Johannesburg district, comprising teams from CMJAH and surrounding similar hospitals (Chris Hani Baragwanath Academic Hospital and Helen Joseph Hospital) have also been set up to champion implementation.

*“The National Policy Framework and Strategy for Palliative Care provides a mandate from the provincial government to facilitate palliative care services in our hospitals”*– **NM2**.

#### Patient needs vs. resources

Management at CMJAH has bought into the integration and routinisation of palliative care in their institution. A medical doctor and three professional nurses have been provided and are fully funded by the hospital to work in the palliative care department. In addition, the assistant nursing manager who is stationed in the Oncology Department under Internal Medicine assists with running the Palliative Care Department. The consensus among management is that palliative care is beneficial for their patients and there are efforts in place to plan for and ensure sustainability.

*“…our matron (assistant manager) attended the strategic meeting we had and then before that she attended our routine meetings. It has opened her eyes to see how important and crucial it is to have palliative care at the hospital. We had not been getting referrals at all*,* in Medical Oncology OPD*,* and she really did assist us with that*,* because now we are getting referrals on a daily basis…”***– CN3**.

## Barriers

### Domain 1: individual characteristics

#### Knowledge, attitudes, and beliefs

From past implementation of palliative care by the previous team, we found that one factor that contributed to the collapse of services was a general lack of awareness of the services that were being offered at the palliative care unit. The previous palliative care team members were usually called when the patients were already too advanced in their illness.

*“… There was a retired nurse who used to assist with palliative care. We used to call her for palliative cases*,* but palliative services at Charlotte were not as great because it was one person you’re calling. And usually*,* we always called when patients were now terminal and needed hospice care…”*– **MD3**.

We also found that no formal palliative care training was received by the other participants who were not part of the palliative care team.

*“So training received was really during the university training*,* but no formal courses or anything. Other training is just via CPD points and those sorts of meetings*,* but no formal training”*– **HD2**.

From the health care providers, we found that while there was some knowledge of palliative care, there was insufficient knowledge of the ideal time for referral to palliative care. We also noted inadequate knowledge of the palliative care screening tool from all participants directly interacting with and providing routine clinical care for patients leading to non-use.

*“There is no formal screening tool that we use and it’s something that I would be excited to look at it and see what we can do and maybe with education and teaching that could be of value”–***HD2**.

We found that prior to establishment of services at CMJAH, there weren’t enough interest in palliative care among relevant stakeholders within the hospital:

*“I don’t think there was interest on it*,* I think if there was interest from the oncologists themselves*,* it would have helped or enabled the service to be there”*– **MD2**.

### Domain 2: intervention characteristics

#### Relative advantage

While participants highlighted the advantages of palliative care services in the hospital, they also noted that there was a high workload for palliative care staff, especially among the social workers and spiritual counsellors. Currently, there is only one social worker, one social auxiliary worker, and two spiritual counsellors working at the palliative care unit and covering the whole hospital. Although efforts have been made to provide staff to work in the palliative care unit, only clinical personnel have been provided. The hospital has not been able to provide staff for other roles such as social workers and spiritual workers and support staff such as clerks and data capturers. This has resulted in a high workload for the existing two personnel providing psychosocial services and two personnel providing spiritual assistance, in addition to having to capture patient data on the palliative care database. Another barrier mentioned was the additional task of screening patients for eligibility for palliative care that is imposed on health care professionals from the different wards.

*“Because now if they were to say the contract (donor funds) have come to an end*,* only two nurses will be left which is currently the permanent staff from Charlotte*,* which means at the end of the day social workers and spiritual counsellors will not be available… so that will be the downfall of palliative care at Charlotte”*– **CN3**.

#### Complexity

Though much sensitization has been conducted in the different hospital wards, the participants noted that most of the referrals they get are for patients who would have already advanced in their disease. This was attributed to the challenges and complexity of training all clinicians from the different wards throughout the hospital. In addition, the current layout of the hospital is such that the palliative care unit is far from most of the wards, especially the oncology wards. This results in patients who would have been referred from the wards not following through with going to the palliative care unit for services.

*“…Palliative care was not within proximity. If we had any psychosocial issues*,* we would have to also refer down to our social workers who were not part of the clinic. As you know*,* the oncology clinic*,* was on the ninth floor. And all these other services were like down in the bottom. So you can never even reliably say if patients went or not. Or they simply just took down a lift and went home…”*– **MD3**.

### Domain 3: implementation process

#### Planning

The personnel currently working in the palliative care unit highlighted that prior to the inception of services in the unit, there was no proper planning for implementation of services. Below is an excerpt from a participant:

*“…as we are running palliative care*,* we’re learning according to our own understanding*,* we don’t have a procedure to say this is how you run palliative care. We’re learning it as we go. I was thrown into the deep end of the ocean*,* and I was told to swim*,* we’ll meet on the other side of the ocean*,* which is really challenging*,* even now it is challenging…”*– **CN1**.

The team also noted that there is no standard for the procedures for referrals, which makes it difficult to follow a systematic approach to providing services in the different wards.

#### Engagement

An issue that was alluded to by most participants was the inadequacy of engagement between palliative care and other disciplines involved in patient care. This was mostly highlighted by non-involvement of palliative care in routine engagements such as academic meetings where clinicians discuss patient cases. The palliative care department is also not part of the routine ward rounds, unlike other specialties such as haematology and microbiology. When asked about possible solutions for this, a participant had the following to say;

*“Members of palliative care team can join in that ward round*,* because that’s when all the other specialties such as microbiology*,* oncology*,* haematology are*,* that’s where all the issues of the patient as a whole are being discussed. So every day they must see the patient. But once a week*,* they must join that combined ward round with the oncologist so that they can hear burning issues and they can also express what they found with the with the patient”*– **MD2**.

### Domain 4: inner setting

#### Implementation climate

Participants alluded to the space allocated for palliative care in CMJAH not being conducive for staff to provide services. Though there has been a space allocated for palliative care, the facilitation of services at the allocated space has not been implemented. This was affected by the fire that destroyed parts of the hospital in April 2021 hence the need for occupational approval for a newly allocated space to be utilised. The allocated space also needs to be renovated. The palliative care staff highlighted that it is difficult to conduct some activities such as family meetings and one-on-one counselling sessions due to inadequate private counselling rooms and inconducive environments for this.

*“…So I think spacing is a big issue here in our hospital*,* we need more space*,* we have a team of at least nine members and we all packed into one room. There’s not enough rooms for us to discuss our patients in a private manner. We have one counselling room. Sometimes patients have to wait because the rooms are being used…”–***CN1**.

Through the interviews, there is a high turnover of doctors, which results in discontinuation of services and the need for retraining when the doctors leave. There is also resistance to opioid prescription by some doctors.

*“…Because doctors are always changing*,* maybe once every six months*,* the palliative care team should have that introduction to inform doctors who they are and that they are available with palliative care…”* -**MD2**.

### Domain 5: outer setting

#### Patient needs vs. resources

While the hospital has managed to provide some clinicians for the palliative care project, they have not been able to sponsor some of the positions that are required for palliative care. We also found that there is no set budget specifically for palliative care at the hospital. The palliative care unit at CMJAH is primarily run through funding from external sources. The project also relies on donor funding for some activities such as hosting support groups, healing services, patient transport, and socio-economic support. In addition to this, the database that is used for storing palliative care patient records is managed through the donor-funded project.

*“… So*,* I think funding is the big elephant in the room. At the end of the day*,* if you don’t have resources*,* you can’t put people in those positions. But I would hope that it’s also seen as a valuable intervention that hopefully can attract funding…”*– **HD3**.

## Discussion

Based on the findings of our study, an interaction of multiple factors can affect the availability and integration of palliative care into routine services at a hospital. Using the CFIR framework, we categorised the identified factors into individual factors, intervention characteristics, the implementation process, inner setting (the setting in which the intervention is implemented), and outer setting (the setting in which the inner setting exists) [17].

Among all participants interviewed for this study, there was some knowledge of palliative care services provided at the hospital. Despite this adequate knowledge of existing services, there is still a challenge of lack of full understanding of what palliative care entails among health care providers, resulting in barriers to availability and integration. The WHO recognises a lack of awareness of what palliative care is among health professionals, policymakers, and the public as a barrier to the availability and integration of services [5]. Study participants also highlighted that they knew of the local policy for palliative care (NPFSPC) that is in place to guide the implementation of palliative care in the province. Without the availability and implementation of policy, and synergies with local authorities, palliative care programs tend to remain vulnerable [22].

Though all personnel from the palliative care unit have received training and have adequate skills to provide palliative care, other health care providers at the hospital have not received formal training. Insufficient palliative care training delays timely referrals to palliative care services while also risking adequate pain management due to healthcare providers’ overly restrictive opioid prescribing practices [5]. In the medical school curriculum for South Africa, students are exposed to case studies that include palliative care during their clinical rotation [23]. However, there may be need for more detailed training particularly for oncology specialists and specialists who manage chronic diseases. While palliative care is increasingly recognized as an essential component of oncology training in South Africa, it has not been uniformly mandated across all medical schools, though significant efforts to integrate palliative care into both undergraduate and post graduate oncology curricula have been made [23].

Officially launching palliative care services before inception provides a foundation for the availability and integration of services in a hospital [24]. From this study, we found that before the inception of the current palliative care project, there was no official launch of services at the hospital. The official launch of services was only done in the second year of implementation. Ideally, during the planning phase for institutional implementation, shared decision-making through consultative meetings, awareness creation, and co-development of standard operating procedures should be done to ensure project success [25]. In a regional development and implementation of the palliative care quality improvement project, planning for project inception was done a year before implementation; where preliminary work included introduction of the project, assessment of available infrastructure, and establishment of operational processes [25]. A similar exercise, where central hospital management, management from selected departments, clinicians, and other allied professionals were engaged well in advance before implementation would have improved the implementation process at CMJAH, leading to better integration into routine services.

The palliative care department at CMJAH currently receives funding from the Bristol Myers Squibb Foundation (BMSF) [14]. Through the BMSF grant, positions that are not part of the Gauteng Department of Health personnel structure, such as spiritual workers, patient navigators, and social auxiliary workers are funded to provide comprehensive services at CMJAH [14]. BMSF also funds roles for other clinical personnel and support and administrative staff [14]. At CMJAH, the service cannot be made available without external funding because currently there is no existing allocated budget for palliative care from the hospital and the Gauteng Department of Health. This finding is congruent with another study that highlights the essentiality of a palliative care budget to support and train healthcare professionals and provide other services [26]. A cost analysis study conducted at a similar hospital setting in South Africa found that the largest costs for a hospital-based palliative care project (63% of total costs) were attributable to personnel costs [6]. The over-reliance on donor funds at CMJAH was noted to have been the major contributor to the collapse of the previous palliative care project at the hospital [14]. Other studies have found that overreliance on donor funds contributes to the poor sustainability of palliative care programs [23, 24]. While it is commendable that the hospital has provided clinical staff for palliative care, it is still very crucial for a set budget for palliative care to be established to ensure that the service provision becomes sustainable.

Consistent with another study on early palliative care consults and their effect on patients’ length of stay [28], stakeholders at the hospital believe that palliative care provides a relative advantage of reduced hospital costs, reduced average length of stay (ALOS), and reduced readmission rates. This perception, coupled with the belief that palliative care improves patient outcomes and quality of life, motivates health care providers and management to ensure implementation of palliative care at the hospital. Several other studies have also highlighted the relative advantage of palliative care in a hospital [23, 24]. Despite some participants noting the advantage of integration and routinisation of palliative care, other health care providers highlighted the additional tasks that come with screening and referrals to palliative care. This was however outweighed by the benefits of palliative care at the hospital. Stakeholder perception of the advantage of an intervention will more likely translate to implementation success [17].

The organizational climate in an institution can either be an enabler or a barrier to efforts for availability and integration of services [32]. While there are high levels of enthusiasm among personnel who are part of the palliative care unit and management, we found that palliative care is not very well supported among other staff and is very individual- driven. Paes et al. identified that a lack of good leadership from senior managers and senior clinicians may lead to poor uptake of palliative care interventions [33]. A high staff turnover, due to the hospital being an academic hospital also negatively impacts the availability and integration of services [34]. Registrars in training are required to go through rotations to ensure different aspects of training are covered. The potential effect on palliative care services is dual– the need for retraining personnel at frequent intervals may be a disadvantage when staff is constantly changing [33]. On the other hand, over time there will be a pool of trained specialists who have gone through the training and are more knowledgeable about the need for palliative care and what it entails. It would also be beneficial if palliative care is incorporated into the medical school curriculum and as part of the required training for oncologists [35].

The space that the palliative care team currently operates in is not conducive to facilitate counselling sessions, family meetings, and clinical consultations. This poses a major setback in effective implementation at the hospital. The importance of a well-thought-out design for a palliative care unit environment has been established [36]. An ideal space for the provision of palliative care clinical and non-clinical services creates a supportive environment for both patients and families [33]. Renovations of the identified space for palliative care at CMJAH must be expedited.

The findings of this study are crucial not only in the context of CMJAH but also at a national and possibly international level. Our study showed that though the policy for palliative care may have been developed in 2017 for South Africa, availability and integration at a tertiary level is not optimal. Other African countries like Malawi, Mozambique, Rwanda, Swaziland, Tanzania, and Zimbabwe have developed their policies [37]. There is, however, a paucity of research indicating the translation of policy into practice in these countries with an active palliative care policy. This research presents opportunity for further research to be conducted on the impact of policy on palliative care availability and integration in tertiary institutions.

### Study limitations

Despite best efforts, we could not secure an appointment with the Chief Executive Officer of the hospital and the management from the Radiation Oncology Department posing a potential of missing some helpful insights to our study. We however managed to interview a consultant in the Radiation Oncology Department who represented management.

## Conclusion and recommendations

While there is a working model for the provision of in-hospital palliative care services at CMJAH, there still stands a crucial need for reduced reliance on external funds to avoid disruption of services. Steps towards sustainability, such as submission of palliative care personnel structures to the GDoH, relationship building with the provincial team, engagement with hospital management, and training of health care professionals for palliative care in the province are most likely to improve implementation. Other internal factors, such as proper planning for palliative care implementation, communication and engagement with other departments, and inclusion of palliative care in academic meetings and during ward rounds will also improve palliative care availability and integration into routine services. There is also an inordinate need for a more conducive space and improved visibility to encourage easier access to services and improve referrals for palliative care.

Currently, the hospital only provides hospital-based outpatient services, however, there is a need to expand and provide palliative care beds and wards in a designated palliative care unit. Management is also encouraged to expedite space allocation and renovation for palliative care and assign other roles such as pharmacists trained in palliative care, data clerks, social workers, and spiritual chaplains towards palliative care.

## Data Availability

Data generated through this research, including interview recordings and transcripts are not publicly available due to a potential lack of anonymity with the interviews and staff roles.

## Abbreviations

AIDS: Acquired Immuno-Deficiency Syndrome
CIFR: Consolidated Framework for Implementation Research
CHBAH: Chris Hani Baragwanath Academic Hospital
CMHPCT: Charlotte Maxeke Hospital Palliative Care Team
CMJAH: Charlotte Maxeke Johannesburg Academic Hospital
DoH: Department of Health
GDoH: Gauteng Department of Health
HJH: Helen Joseph Hospital
HREC: Human Research Ethics Committee
LMIC: Low-and Middle-Income Countries
NDoH: National Department of Health
NPFSPC: National Policy Framework and Strategy for Palliative Care
PC: Palliative Care
PCU: Palliative Care Unit
WHO: World Health Organization
